# Early start of hemoadsorption is associated with improved kidney recovery in ICU patients with ischemic/reperfusion cause of rhabdomyolysis

**DOI:** 10.1371/journal.pone.0352417

**Published:** 2026-06-30

**Authors:** Vedran Premuzic, Alan Horvat, Mate Mogus, Ivona Vucic, Ivan Situm, Tina Tomic-Mahecic, Zeljko Colak, Mirabel Mazar, Robert Baronica, Slobodan Mihaljevic

**Affiliations:** 1 Department of Nephrology, Hypertension, Dialysis and Transplantation, University Hospital Center Zagreb, Zagreb, Croatia; 2 School of Medicine, Catholic University of Zagreb, Croatia; 3 Clinic of Anesthesiology, Resuscitation and Intensive Care, University Hospital Center Zagreb, Zagreb, Croatia; 4 School of Medicine, University of Zagreb, Croatia; Barking Havering and Redbridge Hospitals NHS Trust: Barking Havering and Redbridge University Hospitals NHS Trust, UNITED KINGDOM OF GREAT BRITAIN AND NORTHERN IRELAND

## Abstract

**Background:**

Hemoadsorption is considered currently as an adjunct therapy for the treatment of rhabdomyolysis and acute kidney injury (AKI) although the exact timing is not yet determined. We hypothesised that earlier start of hemoadsorption in patients with ischemia/reperfusion caused rhabdomyolysis and AKI leads to a better clinical improvement and recovery of kidney function.

**Materials and methods:**

We conducted a single-center, retrospective cohort study. Treatment was defined as the use of hemoadsorption with CytoSorb® in combination with continuous renal replacement therapy (CRRT) compared to patients who were treated only with CRRT. Patients were divided in early and late initiation of hemoadsorption subgroups by less or more than 12 hours after developing acute kidney injury. Myoglobin and creatine kinase plasma levels were measured shortly before, 12 hours after the start of treatment, and 24, 48, and 72 hours after initiation. The follow-up lasted until the last enrolled patient reached 60 days after first hemoadsorption procedure.

**Results:**

Overall, 30 patients were included in the treatment arm and 25 patients in the control group. Significant decrease of myoglobin levels was observed in the hemoadsorption treated group in all time points when compared with the control group. Logistical regression analysis found the association of hemoadsorption use and shorter duration of AKI (OR 3.46) and less acute kidney disease (AKD) (OR 2.85). Earlier start of hemoadsorption was associated with a statistically significant shorter duration of AKI (OR 3.22) and less AKD (OR 3.10). Patients treated early with hemoadsorption survived significantly longer than patients treated late (49.2 vs 15.3 days; p < 0.001).

**Conclusions:**

Early start of combined CRRT and hemoadsorption therapy with CytoSorb was safe and associated with improved kidney recovery and survival in patients with ischemic/reperfusion rhabdomyolysis and AKI. Early start of hemoadsorption might prevent renal failure and acute kidney disease and shorten the CRRT dependency in patients who develop AKI.

## Background

The standard treatment for rhabdomyolysis-associated acute kidney injury (AKI) has traditionally been supportive in nature. Severe rhabdomyolysis results in the release of both myoglobin and creatinine kinase (CK) into the bloodstream, which can damage proximal tubule cells in the kidneys [1,2]. As plasma myoglobin levels remain elevated for an extended period, the ongoing injury to the kidneys increases the likelihood of impaired recovery of renal function. Although rhabdomyolysis most commonly develops following mechanical muscle trauma, its etiology may also include various drugs, toxins, infections, muscle ischemia, and electrolyte or metabolic disturbances [3]. Reperfusion additionally triggers an immune response characterized by leukocyte migration into damaged muscle tissue, leading to the release of cytokines, prostaglandins, and free radicals. This process promotes further myolysis and necrosis, potentially resulting in acute kidney injury (AKI) requiring intensive care unit (ICU) admission and multi-organ support [4]. The requirement for kidney replacement therapy in cases of severe rhabdomyolysis is linked to an increased mortality rate among critically ill patients [5,6] but data on the incidence of acute kidney disease (AKD) does not exist. Dialyzers with larger cut-off values have been utilized successfully for several years in clinical practice [7–9]. However, there is a notable lack of data regarding their efficacy in terms of patient outcomes. Another alternative method of removal myoglobin from peripheral blood is by using hemoadsorption. Since the use of CytoSorb has been approved in 2019 for treatment of rhabdomyolysis [10], studies have demonstrated significant reduction of myoglobin in peripheral blood and positive effects in renal recovery [11–14]. CytoSorb is a synthetic adsorption column with surface area of 45,000 m^2^ significantly larger than standard dialysers used for continuous renal replacement therapy (CRRT) [15,16]. Recently published consensus supports the adjuvant hemoadsorption therapy with CytoSorb due to successful removal of circulating myoglobin [17]. Hemoadsorption therapy, whether used alone or in combination with CRRT, may be associated with several potential adverse effects or complications. These include a transient or mild decrease in platelet counts, circuit clotting—particularly in the presence of high circulating myoglobin levels—and the unintended removal of hydrophobic substances such as concomitant medications. Additionally, in cases of ongoing muscle injury, a rebound increase in myoglobin and creatine kinase CK levels may occur, especially when the hemoadsorption cartridge becomes saturated [18]. Hemoadsorption is considered currently as an adjunct therapy and the exact timing is not yet determined although some studies showed that earlier start could have benefits in improving clinical features of patients with sepsis [19,20]. To that end we hypothesised that earlier start of hemoadsorption in patients with ischemia/reperfusion caused rhabdomyolysis and AKI leads to a better clinical improvement and recovery of kidney function.

## Materials and methods

### Study design

We conducted a single-center, retrospective cohort study in the UHC Zagreb intensive care unit (ICU) from April 2021 to February 2025. The study was conducted in accordance with the Declaration of Helsinki. The local institutional review board approved the study (UHC Zagreb, Croatia-number:02/21-JG). Prior to inclusion in the study, written informed consent was obtained from patients or their legal representatives as approved by the review board. The data were accessed for research purposes from March 1^st^ to March 31^st^ 2025. The authors had no access to information that could identify individual participants during or after data collection.

### Inclusion and exclusion criteria

Adult patients (≥ 18 years) with ischemia/reperfusion cause (after aortic surgery or muscle ischemia caused by major artery occlusion) of rhabdomyolysis and the need for CRRT due to anuric/oliguric AKI, diagnosed according to the Kidney Disease Improving Global Outcomes (KDIGO) consensus criteria [21], were included. The indications for urgent aortic surgery were type A aortic dissection, intramural hematoma, or traumatic rupture. Patients in both groups had a longer duration of cardiopulmonary bypass/surgery (exceeding 180–240 minutes) or developed intraoperative hypotension. Causes for major artery occlusion were either thrombosis or dissection of abdominal aorta, iliac or femoral arteries which management involved vascular repair and/or prompt fasciotomy for compartment syndrome. KDIGO currently defines AKI by one of following criteria: serum creatinine (sCr) by ≥0.3 mg/dL within 48 hours, sCr ≥ 1.5 times the baseline value within last 7 days, or urine volume output of ≤ 0.5 mL/kg/h for 6 hours. In addition, patients had to be diagnosed with rhabdomyolysis and plasma myoglobin levels  >  5000 µg/l. Although all patients in both groups were on mechanical ventilation, this was coincidental and not part of the inclusion criteria. The exclusion criterion was no consent from the patient or their legal representatives to participate in the study, due to missing clinical or laboratory values necessary for analysis, patients with polytrauma as a cause for rhabdomyolysis, patients with prior chronic kidney disease (CKD) stage ≥ 3, end-stage renal disease on long-term dialysis or those treated with CRRT before ICU admission were excluded. CKD was defined as abnormalities of kidney structure or function lasting for more than three months, characterized by either a Glomerular Filtration Rate (GFR) ≤ 60 mL/min/1.73/m^2^ or markers of kidney damage like albuminuria, structural abnormalities, or a history of kidney transplant [22]. No sample size estimation was performed in the absence of available data.

### Treatment protocol

Treatment was defined as the use of hemoadsorption in combination with CRRT compared to patients who were treated only with CRRT. The hemoadsorption device was a disposable cartridge (CytoSorb®(Cytosorbents, Manmouth Junction, USA). Hemoadsorption was prescribed for the maximum of 72 hours, or less if patients developed a significant reduction of myoglobin levels (>50–70% reduction), kidney recovery (independence from CRRT) or died during the procedure. The CRRT circuit and the cartridges were replaced every 12–24 hours depending on the effectiveness of the therapy regarding removal rates of myoglobin or indirect signs of cartridge saturation noted by rebound increase in vasopressor levels, high venous pressures on the CRRT machine or clotting. The timing of CRRT circuit and cartridge changes was determined by a study-specific sampling protocol and does not represent standard clinical practice. The indication for hemoadsorption was at the discretion of the attending physician and independent of the study. Regarding the timing of hemoadsorption, patients were divided in early and late initiation of hemoadsorption subgroups by less or more than 12 hours after developing AKI. All patients were classified as stage 3 AKI which we defined as serum creatinine ≥3.0 times baseline, or ≥4.0 mg/dL, or starting renal replacement therapy [23].

The cartridges were added on the PrismaFlex systems (Baxter, IL) with AN69ST-150 filter or the multiFiltrate® CRRT (Fresenius, Germany) with Ultraflux® AV 600 S filter. All CRRT procedures were either continuous veno‐venous hemodiafiltration (CVVHDF) or continuous veno-venous hemodialysis (CVVHD) (15 patients in the hemoadsorption and 12 patients in the control group were treated with CVVHDF). Low-molecular weight heparin (LMWH) anticoagulation was used for all CRRT procedures using a dose protocol based on anti-Xa activity (target 0.25–0.4 U/mL). Regional citrate anticoagulation was not employed in this study owing to its unavailability during the study period. The mean blood flow rate was between 200 and 250 mL/min, depending on blood‐access function and desired ultrafiltration rates. The mean dialysate flow for CVVHD was 2500–3000 mL/h while the mean dialysate and filtrate flow for CVVHDF were 1900–2200 mL/h and 600–800 mL/h. Cytosorb® (CytoSorbents Corporation, Monmouth Junction, NJ, USA). The cartridge was installed pre-dialyzer.

Blood samples (4.5 mL per sample) were collected in serum separator clot activator tubes (Greiner Bio-One GmbH, Kremsmünster, Austria) from the extracorporeal circuit (post-hemoadsorption cartridge, or post-hemofilter during CRRT alone) at predefined time points: 12, 24, 48, and 72 hours after treatment initiation. Furthermore, myoglobin and CK plasma levels (reference ranges were <90 µg/L and 0–153 U/L) were measured shortly before the initiation of treatment. The timing of the blood sampling protocol was determined by a study-specific sampling protocol and does not represent standard clinical practice. CK levels were determined using the ultraviolet photometry while myoglobin levels were determined using an immunoturbidimetric assay. Hemodynamic status was assessed by using blood pressure parameters and the use of vasoactive agents. Patient demographics, comorbidities, laboratory data, and outcome data were from hospital records and ICU charts by clinicians.

### Outcomes

The outcome data included recovery of kidney function, development of acute kidney disease (AKD), safety events (hypotension, coagulation events, haemoglobin depletion and platelet depletion), mortality, ICU stay duration and hospitalization. AKD was defined as acute or subacute damage and/or loss of kidney function for a duration of between 7 and 90 days after exposure to an AKI-initiating event [24]. The follow-up lasted until the last enrolled patient reached 60 days, after first CRRT/CRRT+hemoadsorption procedure, if the survival time was > 60 days, it was recorded as 60 days.

### Statistical analysis

Patients were stratified according to whether they received hemoadsorption and by early and late start with hemoadsorption. Non-parametric continuous variables were summarized as the median and interquartile range and were compared between groups using the Mann-Whitney U Test. Categorical variables were reported as the number and percentage of patients and were compared between groups using the Chi-squared test. Normally distributed variables were presented as means ± standard deviations, and Student´s t-test for independent samples was used to compare the two groups. P-values <0.05 were considered statistically significant. A logistic regression model analysis, using the Walds test, was used to determine the adjusted association between hemoadsorption therapy and CRRT dependency after controlling for confounders which included baseline demographics, vital signs, and interventions including age, gender, urinary output, presence of acute kidney injury, myoglobin and CK plasma levels, and use of vasoactive agents. A logistic regression model was constructed by adding potential confounders to a base model consisting of the two organ supporting modalities, namely EBP therapy and renal replacement therapy. Only those confounders with a likelihood ratio testing p-value of 0.1 or below were retained and added simultaneously to the base model. The outcome variable was coded 1 and an odds ratio>1 indicated improved outcomes such as shorter AKI duration. Survival analysis was done with Kaplan-Meier curves tested with the log-rank test, while hazard ratios were estimated with Cox proportional hazards regression. Multiple linear regression was used to explore the influence of different variables on CRRT dependency. Statistical analysis was performed using SPSS version 23.0 (IBM Corp., USA).

## Results

### Baseline characteristics

**Fig 1:**
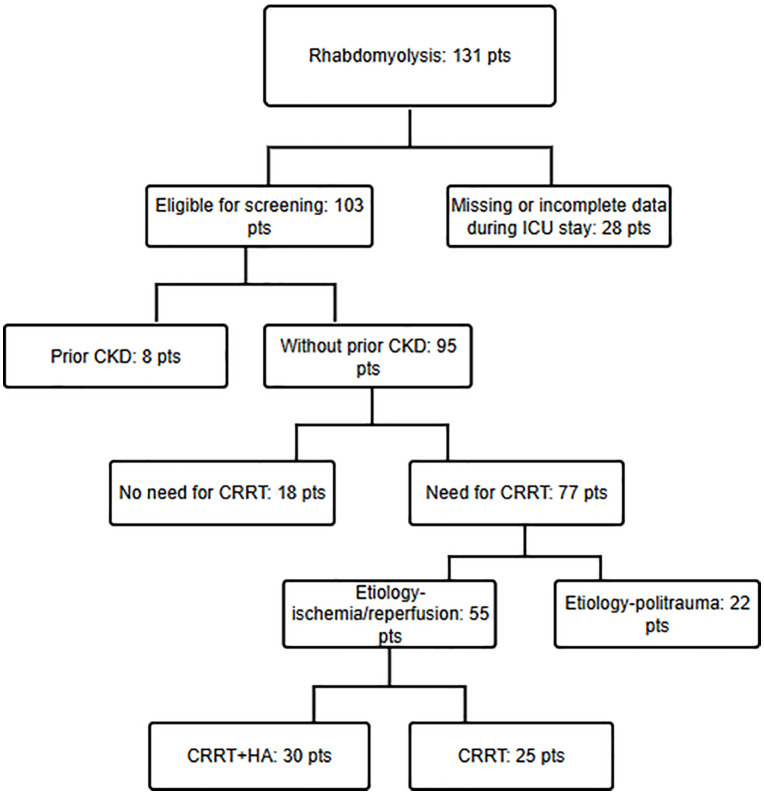
A patient flowchart. ICU-intensive care unit; CKD-chronic kidney disease; CRRT-continuous renal replacement therapy; HA-hemoadsorption.

Fig 1 illustrates the selection of the study population based on the above-mentioned inclusion and exclusion criteria. Overall, 30 patients were included in the treatment arm and 25 patients in the control group.

At baseline, there were no differences in age, gender, number of patients on vasoactive support, dose of vasopressors and urinary output between these two subgroups of patients (**Table 1**).

**Table 1:** Demographic parameters and differences in clinical variables and laboratory parameters between patients with and without hemoadsorption at baseline.

Variables	Hemoadsorption (N = 30)	Controls (N = 25)	p
Age (years)	56.9 + 3.5	56.3 + 3.0	0.89
Sex (males) N(%)	21 (70.0)	15 (60.0)	0.43
BMI	27.4 + 2.6	27.5 + 2.9	0.89
Etiology of Rhabdomyolisis N(%)			
*After aortic surgery*	14 (46.6)	12 (48.0)	0.92
*Muscle ischemia due to major artery occlusion*	16 (53.4)	13 (52.0)	
Mechanical ventilation N (%)	30 (100)	25 (100)	0.99
Acute kidney injury stage 3 N (%)	30 (100)	25 (100)	0.99
Hemodynamic status			
Mean arterial pressure (mmHg)	49.82 + 4.3	49.88 + 4.3	0.98
All Vasopressors N (%)	25 (83.3)	20 (80.0)	0.75
*Norepinephrine*	20 (66.6)	18 (72.0)	0.66
*Vasopressin*	14 (46.6)	12 (48.0)	0.92
*Epinephrine*	6 (20.0)	4 (16.0)	0.70
Vasoactive therapy dose mcg/kg/min	0.67 + 0.01	0.64 + 0.01	0.82
APACHE IV	71 (43-97)	72 (45-99)	0.76
Urinary output (mL/h)	11 (0-32)	7 (0-20)	0.47
Laboratory data			
Lactates (mmol/L)	8.1 + 0.93	6.7 + 0.77	0.47
pH	7.23 + 0.84	7.26 + 0.87	0.15
Potassium (mmol/L)	4.98 + 0.32	4.79 + 0.25	0.24
INR	1.43 + 0.06	1.23 + 0.06	0.15
Platelets (x10^9^/L)	88 (44-136)	97 (56-153)	0.73
Creatinine (µmol/L)	191.8 + 23.2	197.6 + 24.9	0.86
BUN (mmol/L)	13.3 + 1.8	15.2 + 1.9	0.45
C-reactive protein (mg/L)	137 (76-212)	186 (122-264)	0.11
Procalcitonin (µg/L)	6.04 + 0.93	7.37 + 0.98	0.14
Creatine kinase (U/L)	26568 (17812-35745)	28912 (19823-37233)	0.64
Myoglobin (µg/L)	63542 (48722-77933)	43721 (29012-57234)	0.21

BMI-body mass index; INR-international normalized ratio GFR-estimated glomerular filtration rate; BUN-blood urea nitrogen; results are shown as mean + /- SD or median (interquartile range)

Both patient groups had similar levels of lactates, C-reactive protein (CRP), CK and myoglobin (all p > 0.05). No treatment-specific complications such as the development of severe thrombocytopenia, severe bleeding, thromboembolism, or electrolyte disorders were observed. In 5 patients where the cause of rhabdomyolysis was not solved (i.e., compartment syndrome without surgical intervention) we have observed prolonged high levels of both CK and myoglobin.

### Differences in Clinical Variables and Laboratory Data between patients with and with-out hemoadsorption

The overtime variation of clinical and laboratory data observed for the hemoadsorption and control groups before, during and after treatments are presented in Table 2. The mean duration of EBP was 52.5 hours. At 48 hours after start of CRRT there was a significantly lower numbers of patients on vasopressors, lower lactate levels and a significant increase in urinary output in the group treated with hemoadsorption (all p < 0.05).

**Table 2:** Intra-group overtime variation differences between hemoadsorption and control group of patients.

Hemoadsorption						Control				
	before HA+CRRT	12h after start of HA+CRRT	24h after start of HA+CRRT	48h after start of HA+CRRT	72h after start of HA+CRRT	Before CRRT	12h after start of CRRT	24h after start of CRRT	48h after start of CRRT	72h after start of CRRT
Vasoactive therapy (N(%))	25 (83.3)	**16 (53.3)** ^**#**^	**12 (41.4)** ^**#**^	**8 (26.6)** ^**#**^	**5 (16.6)** ^**#**^	20 (80.0)	**13 (52.0)** ^**#**^	**12 (48.0)** ^**#**^	**13 (52.0)** ^**#**^	**8 (32.0)** ^**#**^
Urinary output (mL/h)	11 (0-32)	11 (0-32)	**23 (0-44)** ^**#**^	**38 (5-66)** ^**#**^	**51 (10-88)** ^**#**^	7 (0-20)	7 (0-20)	8 (0-25)	11 (0-40)	21 (5-60)
Lactates (mmol/L)	8.1 + 0.9	7.4 + 0.3	**5.3 + 0.2** ^**#**^	**4.2 + 0.2** ^**#**^	**3.4 + 0.2** ^**#**^	6.7 + 0.7	8.9 + 0.8	9.1 + 0.9	9.4 + 0.9	9.5 + 0.9
CK (U/L)	26568 (17812-35745)	26431 (17698-35656)	**20388 (11476-296301)** ^**#**^	**16503 (8576-24689)** ^**#**^	**16199 (8255-24314)** ^**#**^	28912 (19823-37233)	27577 (18412-36404)	27004 (19344-36767)	26790 (19189-37008)	20218 (12779-30904)
Myoglobin (µg/L)	63542 (48722-77933)	**46877 (32801-60003)** ^**#**^	**32517 (19774-45930)** ^**#**^	**20119 (7877-33788)** ^**#**^	**18662 (6893-31004)** ^**#**^	43721 (29012-57234)	42533 (28554-56711)	40198 (26644-54203)	38824 (25112-52209)	33101 (19892-47220)
AKI stage 3 Yes (N(%))	30 (100)	27 (90.0)	**25 (83.3)**	**19 (63.3)** ^**#**^	**14 (46.6)** ^**#**^	25 (100)	2 (92.0)	2 (92.0)	20 (80.0)	**16 (64.0)** ^**#**^

HA-hemoadsorption; CRRT-continuous renal replacement therapy; CK-creatine kinase; AKI-acute kidney injury; results are shown as mean + /- SD or median (interquartile range); ^# -^ p < 0.05 for intra-group variation.

**Fig 2:**
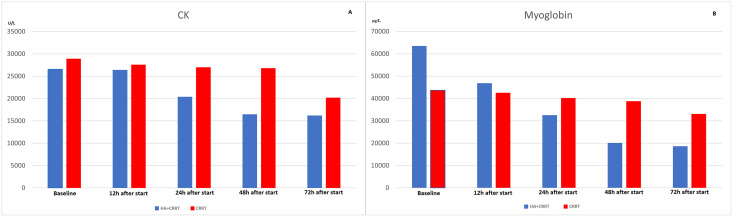
Differences in the course of CK (A) and myoglobin (B) prior and during therapy between patients treated with the combination of HA and CRRT and patients treated with CRRT. CK-creatine kinase; CRRT-continuous renal replacement therapy; HA-hemoadsorption.

Significant decrease of myoglobin levels was observed in the hemoadsorption treated group in all time points when compared with the control group (all p < 0.01) while the removal rates for CK were higher on the 24h and 48h after starting with therapy in the hemoadsorption group (both p < 0.05) but with similar removal rates at 72h after starting with the procedure (p > 0.05) (Fig 2A and 2B). We have not found differences in myoglobin or CK removal between different CRRT modalities in both hemoadsorption and control group of patients.

### Differences in Clinical Variables and Laboratory Data between Patients Treated with early and late start of hemoadsorption

At baseline, patients treated with late start of hemoadsorption were almost significantly older (p = 0.06) and had higher number of patients on vasopressors when compared with patients with early start of hemoadsorption (p = 0.03) (**Table 3**).

**Table 3:** Demographic parameters and differences in clinical variables and laboratory parameters between patients with early and late start of hemoadsorption.

Variables	Early (N = 17)	Late (N = 13)	p
Age (years)	52.7 ± 3.1	62.4 ± 3.9	0.06
Sex (males) N(%)	12 (70.6)	9 (69.2)	0.77
BMI	26.4 ± 2.7	28.2 ± 3.0	0.59
Etiology of Rhabdomyolisis N(%)			
*After aortic surgery*	7 (41.2)	6 (46.1)	0.78
*Muscle ischemia due to major artery occlusion*	10 (58.8)	7 (53.9)	
Mechanical ventilation at baseline N (%)	17 (100)	13 (100)	0.99
Acute kidney injury stage 3 at baseline N (%)	17 (100)	13 (100)	0.99
Number of cartridges per patient	2.6 ± 0.02	2.9 ± 0.02	0.63
Overall duration of hemoadsorption (hours)	50.34 ± 3.0	52.25 ± 3.4	0.77
Hemodynamic status			
Mean arterial pressure (mmHg)	57.69 ± 5.8	48.50 ± 4.1	**<0.01**
All Vasopressors at baseline N (%)	12 (70.6)	13 (100)	**0.03**
*Norepinephrine*	12 (70.6)	8 (61.5)	0.60
*Vasopressin*	7 (41.2)	7 (53.8)	0.49
*Epinephrine*	3 (17.6)	3 (23.1)	0.71
Vasoactive therapy dose at baseline mcg/kg/min	0.38 ± 0.01	0.94 ± 0.01	0.16
APACHE IV	72 (45-96)	78 (49-101)	0.13
Urinary output at baseline (ml/h)	11 (0-30)	5 (0-18)	0.33
Laboratory data			
Lactates (mmol/L)	4.3 ± 0.52	6.1 ± 0.73	0.39
pH	7.24 ± 0.84	7.25 ± 0.89	0.65
Potassium (mmol/L)	4.96 ± 0.30	4.84 ± 0.29	0.38
INR	1.39 ± 0.05	1.44 ± 0.08	0.41
Platelets (x10^9^/L)	89 (40-132)	88 (40-128)	0.84
Creatinine (umol/L)	190.3 ± 23.0	193.5 ± 24.2	0.77
BUN (umol/L)	13.1 ± 1.7	13.8 ± + 1.9	0.53
C-reactive protein (mg/L)	133 (71-209)	157 (104-232)	0.49
Procalcitonin (µg/L)	5.96 + 0.91	6.88 + 0.95	0.13
Creatine kinase (U/L)	24152 (16224-32006)	29727 (20987-37541)	0.59
Myoglobin (µg/L)	37737 (25368-50101)	97288 (75877-117989)	0.21
Outcomes			
Acute kidney injury at the end of ICU stay N (%)	2 (11.7)	12 (92.3)	**<0.001**
AKD N (%)	2 (11.7)	11 (84.6)	**<0.001**
CRRT dependancy days	5.3 ± 0.88	12.7 ± 1.44	**0.04**
ICU survival days	30 (4-57)	14 (2-24)	**<0.001**

BMI-body mass index; INR-international normalized ratio GFR-estimated glomerular filtration rate; BUN-blood urea nitrogen; AKI-acute kidney injury; AKD-acute kidney disease; CRRT-continuous renal replacement therapy; ICU-intensive care unit; results are shown as mean + /- SD or median (interquartile range)

Both patient groups had similar levels of lactate, C-reactive protein (CRP), CK and myoglobin. In the group with early start of hemoadsorption we have found significantly shorter CRRT dependency (p = 0.04) and longer ICU survival in days (<0.001), and significantly smaller number of patients with AKI and AKD at the end of follow-up (both p < 0.001). We have found similar rates of both CK and myoglobin removal in all time points between patients treated with early and late start of hemoadsorption.

In the linear regression analysis in the entire cohort, CRRT dependency was independently and negatively associated with hemoadsorption (β = −0.269, p = 0.048) while interestingly age and CK and myoglobin levels were not (Table 4). In the additional model in the hemoadsorption group of patients, CRRT dependency was independently and negatively associated with the early start of hemoadsorption (β = −1.138, p = 0.033).

**Table 4:** Associations Between Covariates and CRRT dependency.

Model	B	Std. Error	Beta	t	Sig.
**All patients**					
(Constant)	7,908	12,103		0,653	,517
Age	,309	,400	,113	,744	,443
Gender Males	3,806	2,584	,204	1,473	,148
Hourly diuresis	-,127	,069	-,271	−1,827	,074
Vasoactive therapy	,015	,014	,150	1,075	,288
Lactates	,331	,234	,244	1,417	,163
CK	,000	,000	,015	,067	,947
Myoglobin	,000	,000	,017	,089	,930
Hemoadsorption	−4,801	2,364	-,269	−2,031	**,048**
**Hemoadsorption patients**					
Model	B	Std. Error	Beta	t	Sig.
(Constant)	1,074	11,044		,097	,923
Age	,117	,161	,181	,726	,476
Gender Males	4,096	4,355	,195	,941	,358
Hourly diuresis	-,067	,100	-,122	,670	,511
Vasoactive therapy	5,149	5,696	,200	,904	,377
Lactates	,796	,927	,044	,267	,711
CK	,000	,000	,202	,726	,476
Myoglobin	,000	,000	,452	1,550	,137
Early start of Hemoadsorption	−22,327	9,766	−1,138	−2,286	**,033**

Dependent Variable for All patients and for Hemoadsorption patients: CRRT dependancy days.

CK-creatine kinase; CRRT-continuous renal replacement therapy.

### Variables associated with improved kidney function

Logistical regression analysis using the Walds test in the entire cohort found the association of hemoadsorption use and shorter duration of AKI in days (odds ratio 3.46, 95% confidence interval 2.22–4.60) and less AKD (odds ratio 2.85, 95% confidence interval 1.68–4.12). In subgroup analysis regarding timing of hemoadsorption, earlier start of hemoadsorption was associated with a statistically significant shorter duration of AKI in days (odds ratio 3.22, 95% confidence interval 2.68–3.90) and less AKD (odds ratio 3.10, 95% confidence interval 2.22–4.02) while hemoadsorption treatment duration and number of cartridges did not had any significant association.

### Patient survival

**Fig 3:**
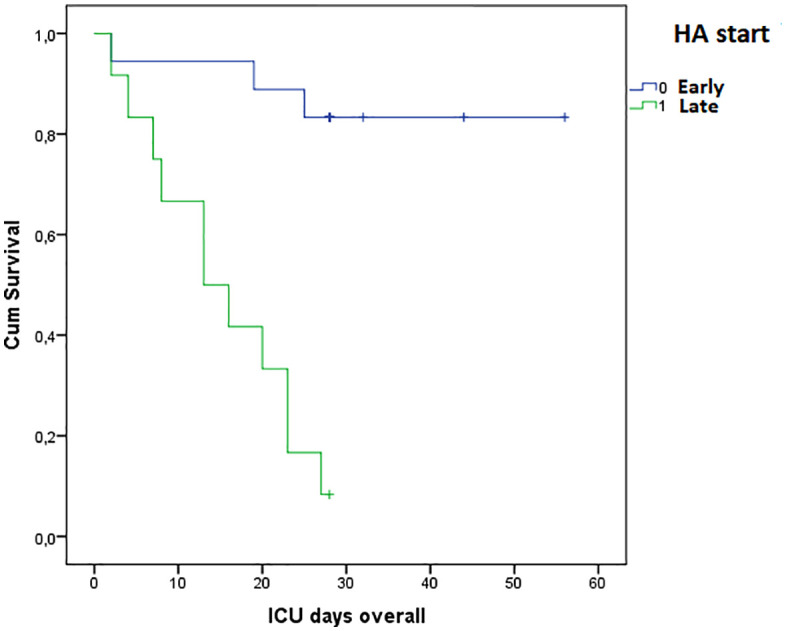
Kaplan-Meier analysis of survival probability according to early versus late start of HA. HA-hemoadsorption; ICU-intensive care unit.

Twenty seven patients (49%) died before 60-days of follow-up, 14 (46.6%) in the hemoadsorption group, 13 (52.0%) in the control group. We have not found differences in the mean survival time between these two groups of patients (36.6 [95% CI 28.9, 44.3] vs 36.0 [95% CI 28.2, 43.7] days; p > 0.05). Patients treated early with hemoadsorption survived significantly longer than patients treated late (49.2 [95% CI 41.9, 56.5] vs 15.3 [95% CI 10.5, 20.2] days; p < 0.001) (Fig 3). A delayed start of hemoadsorption was associated with higher mortality (HR 1.02 [1.00, 1.04], respectively).

## Discussion

This study represents the first report evaluating the effects of hemoadsorption in patients with ischemia–reperfusion caused rhabdomyolysis and AKI. The results suggested that early start of hemoadsorption therapy with CytoSorb was associated with improved kidney recovery and survival in ICU patients with rhabdomyolysis and AKI. Hemoadsorption plus CRRT therapy, when compared to standard CRRT therapy, was also associated with a decrease in use of vasoactive therapy and reduced lactate levels and an increase in urinary output. Our findings are underlining the importance of early start like stated in the consensus paper [17].

Recent studies have reported that the combination of CRRT with hemoadsorption can effectively remove myoglobin and CK [14,25] but most of studies have showed that myoglobin concentration decreased more rapidly [13,25]. This is in agreement with our study findings, in which we observed lower removal rates of CK compared with myoglobin following hemoadsorption, both after the procedure and when compared with the control group. The results from the study by Zhou et al. [26] showed the significant decrease in CK levels at 24 and 48h which is consistent with our results. Nevertheless, the use of medium cut-off filters (e.g., EMIc2) may represent an effective alternative for enhancing myoglobin clearance and the removal of other target molecules, without incurring the additional costs and risks of hemoadsorption, including filter clotting and the unintended depletion of platelets and medications [27]. Regarding the timing of hemoadsorption we have found similar rates of both CK and myoglobin removal at all time points. Our study showed that, compared with standard CRRT, hemoadsorption combined with CRRT was associated with improved clinical outcomes, including reduced vasoactive support, lower lactate levels, increased urine output, and faster renal recovery, reflected in shorter CRRT duration and a significantly reduced incidence of AKI (OR 3.46). To our knowledge, this is the first study which investigated the smaller incidence of AKD at the end of follow-up in patients with AKI and rhabdomyolysis treated with hemoadsorption with an OR of 2.85. Study by Scharf et al [14], suggested that the effect of hemoadsorption might be obscured if the cause of rhabdomyolysis is not solved which we have observed in our patients as well. Early initiation of hemoadsorption therapy has been shown to be beneficial in clinical settings, particularly in conditions characterized by the rapid release of toxins or inflammatory mediators, such as sepsis, cytokine release syndrome, or rhabdomyolysis [16,19,28]. Starting hemoadsorption early can reduce circulating levels of systemic inflammatory responses, leading to improved hemodynamic stability which we observed in our study when starting with hemoadsorption in the first 12 hours. There is no universally accepted 12-hour cut-off in the prior literature for defining “early” versus “late” initiation of hemoadsorption in patients with rhabdomyolysis and AKI. While some reports and consensus papers have suggested potential benefit with early initiation (e.g., within 12–24 hours), particularly in septic patients or those with rhabdomyolysis and AKI [17,29], these thresholds are neither standardized nor consistently validated. The selection of a 12-hour cut-off in the present study was therefore only partially inspired by the concept of “early intervention.” An additional rationale was based on biological and clinical considerations, specifically the potential benefit of early myoglobin removal to prevent sustained renal injury, given that peak myoglobin levels are reported to occur approximately 4–12 hours after the initial insult [30]. In the context of conditions like rhabdomyolysis, early hemoadsorption combined with CRRT can enhance the removal of myoglobin and CK, helping to protect kidney function and accelerate recovery. Our results showed that early initiation of hemoadsorption was associated with shorter CRRT dependence and a significantly lower incidence of both AKI (OR 3.22) and AKD (OR 3.10). Nevertheless, this association should be taken cautiously due to significantly older age and higher number of patients on vasopressors and lower mean arterial pressure in the group with late start of hemoadsorption. These differences suggest that patients in the late group may have been more severely ill, which could influence survival and kidney recovery outcomes independently of treatment timing. Interestingly, overall duration of treatment and number of used cartridges was not associated with better kidney recovery and outcome. Therefore, early initiation of hemoadsorption in patients with rhabdomyolysis may be a key determinant in optimizing therapeutic efficacy. To best of our knowledge this is the first study which analyzed a cohort of patients with ischemia-reperfusion events after vascular surgical procedures related to development of rhabdomyolysis and AKI. Understanding the condition of rhabdomyolysis caused by ischemia-reperfusion [31–33] is crucial for timely intervention to prevent renal failure, multi-organ failure and to improve survival. Delayed initiation of hemoadsorption was not only associated with poorer renal outcome but also with higher mortality with a HR of 1.02. Still, larger studies and a prospective randomized controlled trial is needed to confirm the importance of timing of hemoadsorption in rhabdomyolysis associated AKI.

### Limitations

Due to the retrospective and single-center data analysis study approach, the present work has several limitations. The sample size of both patients treated with hemoadsorption and of control group of patients is relatively small and no formal sample size or power calculation was performed which limits the reliability of multivariate analyses and increases the possibility of type I error. However, it must be noted that no study concerning the specific ischemic-reperfusion aetiology of rhabdomyolysis treated with hemoadsorption with this sample size is available to date and the underlying cause of rhabdomyolysis were similarly distributed in both groups. In addition, the observation period was limited to 60 days; it is unclear whether there was renal recovery in some patients later. CK and myoglobin levels were measured in routine clinical practice and not under controlled study conditions. We did not measure pre- and post-cartridge values of CK and myoglobin. Therefore, we were unable to analyze the adsorption and saturation kinetics of the cartridge. Furthermore, control over time of blood solutes was done in samples drawn from post-cartridge points, which can possibly overestimate total body removal and add further bias. The indication for hemoadsorption was determined by the attending physician rather than predefined criteria, which introduces potential selection bias. This may have affected which patients received early versus delayed therapy. The unavailability of citrate anticoagulation was a limitation, as citrate is associated with improved outcomes and reduced circuit clotting, particularly in patients with polytrauma. Additionally, it should be noted that the sample size of this study is too small, and the results of multi-factor analysis may not be reliable.

## Conclusions

Early start of combined CRRT and hemoadsorption therapy with CytoSorb was safe and may be associated with improved kidney recovery and survival in patients with ischemic/reperfusion rhabdomyolysis and AKI. Early start of hemoadsorption might prevent renal failure and acute kidney disease and shorten the CRRT dependency in patients who develop AKI. To improve risk assessment strategies and optimize patient care in the context of this complex condition, further research is warranted.

## Abbreviations

AKI: acute kidney injury
AKD: acute kidney disease
CK: creatinine kinase
CRP: C-reactive protein
CRRT: continuous renal replacement therapy
ICU: intensive care unit
